# Excelsior

**Published:** 1889-12

**Authors:** H. H. Harrison

**Affiliations:** Wheeling, W. Va.


### Excelsior.

BY DR. H. H. HARRISON, WHEELING, W. VA.

Read before the Ohio State Dental Society at Cleveland, October, 1889.

Excelsior is a station of high degree, attainable by many in all professions, but not by every one. It is not a degree of definite limit, but one toward which all our efforts should tend. Those with natural gifts may reach it, and even with most of these it requires energy and application; but with those who may not have natural gifts in a specific line, excelsior can not be reached, though they may struggle never so hard. This latter class is the dead wood that all professions must carry, but in order to place our record before the world in the proper light, we must not only have dental education well in advance, laws also that protect the unwary from the fingers of the quack, but we must adopt some systematic plan of educating the people up to the standard of highest appreciation of the educated dentist. Am glad to say this is being done to a considerable extent by many in the profession, both by precept and example, but it is too slow for this rapid age, and we must have a little more electricity and gas administered to bring about this desired result. I am very sure the best educated talent in our profession to-day is not fully appreciated by the people.

The question may arise in the minds of some, "How can this popular education be brought about?" It will be said by others that there are treatises on popular dentistry before the public, and what benefit have they brought about? They have done good, but are circumscribed in their usefulness by the fact that mercenary motives are too prominent in this production, and too single-handed—not enough authority behind them.

Some two years ago I suggested to some of the members of this society the wisdom of publishing a treatise on Popular Dentistry, to be approved by the society and circulated among our patients without cost to them to be sure to get them into their hands. Of course the publishers would furnish to purchasers, and the State Society would be at no expense except to furnish the brains to write these few pages.

I am well aware that many of you have a business that may not require this so far as you are individually concerned, but how about your patients who are neglecting certain operations, that when pointed out to them they admit that they were totally ignorant of the real facts as they now appear. You can not go into detail with all your patients, for your time is too precious, and this plan will save many a lengthy dissertation on some special point in practice.

When we look around us and discover the numberless cases of poor dentistry tolerated by even intelligent people, because they know no better, it should bring to us serious reflection as to who is responsible for all this. You may talk about the survival of the fittest, but this will permit generations yet unborn to suffer the effects of ignorance, and go down to an untimely end, thinking that dentistry is nothing but a trade and possibly a poor one at that. Give the people to know that there are educated men in our profession that understand the application of remedies for the treatment of pathological conditions that arise within the mouth, better than the general practitioner of medicine. I am perfectly disgusted with the idea that M. D.'s are called to patients with fractured jaws, with alveolar hemorrhage, with tumors of the mouth, hare-lip and ptyalism. This should not be, but until people know more of the sphere of the worldly dentist it must continue. The plan I mention will not only correct such as the above, but it places the quack dentist just where he ought to be, that all may know him by his foot-prints.

This plan has been adopted by our State Society already, and I should like to see the Ohio State Dental Society appoint a committee that would do this work and let the Board of Directors endorse it when submitted to them if it be up to their idea.

Gentlemen, I do not write this without the expectation of criticism, for I know that objections can be found, but what good project can be presented that is free from weakness? But I feel the necessity of this so very much that I can not refrain from speaking out. "Out of the fullness of the heart the mouth speaketh."

